# Management of the Post-Extraction Socket with Buccal Dehiscence in the Esthetic Zone Associated with Immediate Implant Placement Using L-PRF and CTG for Periosteal Inhibition: A Case Report

**DOI:** 10.3390/dj14070445

**Published:** 2026-07-16

**Authors:** Giuseppe Balice, Matteo Serroni, Alessio Frisone, Stefania Di Gregorio, Mauro Di Berardino, Giacinto Di Placido, Corrado Cesaretti, Giovanna Murmura, Michele Paolantonio

**Affiliations:** 1 Department of Innovative Technologies in Medicine and Dentistry, G. d’Annunzio University of Chieti–Pescara, Via dei Vestini 31, 66100 Chieti, Italy; matteo.serroni@phd.unich.it (M.S.); alessio.frisone@phd.unich.it (A.F.); stefania.digregorio@unich.it (S.D.G.); mauro.diberardino@unich.it (M.D.B.); giacinto.diplacido@libero.it (G.D.P.); corrado.cesaretti@studenti.unich.it (C.C.); giovanna.murmura@unich.it (G.M.); mpaolantonio@unich.it (M.P.); 2 Department of Periodontics and Preventive Dentistry, University of Pittsburgh School of Dental Medicine, Pittsburgh, PA 15213, USA

**Keywords:** alveolar ridge preservation, buccal dehiscence, L-PRF, connective tissue graft, CTG, immediate implant placement

## Abstract

**Background:** Management of post-extraction sockets with buccal dehiscence in the esthetic zone remains clinically challenging, particularly when immediate implant placement is indicated. Conventional approaches often rely on guided bone regeneration (GBR) with biomaterials, which may increase surgical complexity and morbidity. This case report evaluates the clinical and radiographic outcomes of a fully autologous approach combining leukocyte- and platelet-rich fibrin (L-PRF) and connective tissue graft (CTG) in conjunction with immediate implant placement. **Methods:** A 50-year-old healthy patient presenting with a fractured maxillary lateral incisor and buccal bone dehiscence underwent atraumatic extraction, immediate implant placement, and simultaneous site management using L-PRF membranes and CTG. Clinical and radiographic evaluations were performed at baseline (T0) and after 12 months (T1). Cone-beam computed tomography (CBCT) was used to assess horizontal bone thickness (HBT) at multiple apico-coronal levels and vertical evaluation parameters, including nasal floor–crest distance (NF–AC). Clinical outcomes included probing pocket depth (PPD), keratinized tissue width (KT), gingival thickness (GT) and patient-reported outcome measures (PROMs). **Results:** Radiographic analysis demonstrated increased HBT at all levels (+1.4 mm at 9 mm, +3.1 mm at 12 mm, +3.2 mm at 15 mm, and +2.6 mm at 18 mm). NF-AC showed a marked increase (+2.7 mm) and substantial reduction in the buccal dehiscence defect. Clinically, KT increased by +2.0 mm and GT by +1.8 mm. Healing was uneventful, with minimal postoperative discomfort and high patient satisfaction. **Conclusions:** Within the limitations of a single case report, the combined use of L-PRF and CTG with immediate implant placement was associated with favorable clinical and radiographic outcomes. These preliminary findings suggest that a fully autologous, biologically driven approach may represent a potential alveolar ridge preservation strategy and a biomaterial-sparing alternative to conventional GBR in carefully selected cases. Further controlled studies are required to confirm these observations and define the indications and limitations of this approach.

## 1. Introduction

Immediate implant placement following tooth extraction represents a widely adopted clinical strategy aimed at reducing overall treatment time and preserving tissue volumes [1].

However, this approach presents significant challenges, particularly in anterior regions with high esthetic demands, where even minimal alterations of the alveolar bone contour and soft tissue architecture can compromise the functional and esthetic outcome of the final restoration [2,3]. After extraction, alveolar bone resorption occurs rapidly and substantially, resulting in a loss of ridge volume that can jeopardize both implant stability and the esthetic profile [2,4].

The presence of a buccal osseous dehiscence represents an additional clinical challenge, as immediate implant placement in such defects may compromise the predictability of bone regeneration and long-term peri-implant stability [5].

From an implant–periodontal perspective, in this case the management of peri-implant soft tissues represents a critical scenario. Recent studies have shown that the quality and quantity of peri-implant keratinized mucosa are strongly correlated with the risk of mucositis and peri-implantitis, directly influencing the biological stability of the implant and the final esthetic outcome [6,7]. In particular, the absence of at least 2 mm of peri-implant keratinized tissue has been associated with higher levels of inflammation, increased marginal bone loss, and greater plaque accumulation [8].

To address the challenges associated with post-extraction ridge resorption, guided bone regeneration (GBR) techniques have been widely adopted in clinical practice. These techniques typically employ resorbable or non-resorbable membranes in combination with xenogeneic or synthetic biomaterials, with the aim of promoting new bone formation by excluding soft-tissue ingrowth and maintaining a protected regenerative compartment [3,9]. However, GBR is not without limitations: the procedures can be technically demanding, costly, and associated with risks such as membrane exposure and postoperative complications. In addition, the use of non-autologous biomaterials may influence the dynamics of wound healing and tissue integration [10].

In this context, the use of autologous biological materials has gained increasing interest. Particular emphasis has been placed on platelet concentrates, such as leukocyte- and platelet-rich fibrin (L-PRF).

L-PRF is a second-generation platelet concentrate characterized by a three-dimensional fibrin matrix that entraps platelets and leukocytes, capable of releasing a variety of growth factors and cytokines—including TGF-β, PDGF, VEGF, EGF, IGF-1, IL-1β, IL-4, IL-6, and TNF-α. These biological mediators may promote neoangiogenesis, stimulate fibroblast proliferation, and enhance epithelial cell migration, thereby facilitating tissue healing. Such properties result in accelerated wound closure, more effective hemostasis, and faster, esthetically favorable scar remodeling [11].

It is now well established that the use of L-PRF enhances both hard and soft tissue healing and regeneration through the gradual release of growth factors [12,13]. However, despite these biological properties, L-PRF membranes present an inherent limitation: their relatively rapid resorption may reduce their ability to stabilize the clot and protect the regenerative site over the medium term [14].

To overcome this limitation and to ensure the preservation of the peri-implant soft tissues, the addition of a connective tissue graft (CTG) harvested from the palate can serve as a biological membrane, stabilizing the soft tissue contour, increasing vestibular thickness, and improving the quality of peri-implant mucosa [6,7].

In parallel, a recently introduced approach—the Periosteal Inhibition (PI) technique—has shifted the focus from promoting bone regeneration to preventing bone resorption [15]. This technique is based on the biological concept that post-extraction bone loss is largely mediated by osteoclast precursor cells originating from the periosteum. By placing a high-density polytetrafluoroethylene (d-PTFE) membrane between the periosteum and the buccal bone plate, the migration of these precursor cells is physically inhibited, thereby reducing their differentiation into mature osteoclasts and limiting osteolytic activity on the external bone surface. As a result, alveolar ridge dimensions may be preserved without the need for heterologous bone grafting materials, representing a biologically distinct strategy for post-extraction site management [15].

Taken together, these considerations provide the rationale for combining L-PRF membranes and CTG within a fully autologous protocol.

Although the use of L-PRF in association with immediate implant placement has already been investigated [16], the novelty of the present case report lies in the application of an adjunctive CTG based on the biological rationale of PI. In this approach, the CTG was used not only for soft-tissue augmentation, but also as an autologous interpositional layer between the periosteum and the alveolar crest, whereas L-PRF membranes were intended to support clot stabilization, angiogenesis, and early wound healing.

Building on this concept and on the surgical technique described for PI by Nguyen et al. [15], the aim of the present case report was to evaluate the clinical and radiographic outcomes of a fully autologous approach combining L-PRF membranes and CTG with immediate implant placement in a post-extraction site presenting buccal dehiscence in the esthetic zone. Postoperative healing, implant survival, regenerated bone-volume stability, and soft-tissue changes, including keratinized tissue width (KT) gingival thickness (GT) and probing pocket depth (PPD) were assessed through clinical follow-up and cone-beam computed tomography (CBCT) at 12 months.

## 2. Materials and Methods

### 2.1. Study Setting and Ethical Considerations

The present clinical case was managed at the Unit of Periodontology and Oral Hygiene, University “G. D’Annunzio” of Chieti-Pescara (Italy). All procedures were performed in accordance with the ethical principles of the Declaration of Helsinki and written informed consent was obtained from the patient for the scientific and educational use of clinical data.

The case report was prepared in accordance with the CARE guidelines.

### 2.2. Clinical Presentation, Case Management, and Outcomes

A 50-year-old male patient, non-smoker, in good general health (American Society of Anesthesiologists classification I), with excellent oral hygiene, a stable periodontal status, and no history of systemic or local conditions impairing bone or soft tissue healing, presented with Grade II mobility of tooth 1.2 associated with a periapical radiolucency and vertical root fracture. Extraction of tooth 1.2 followed by immediate implant placement and simultaneous bone regeneration was planned. Clinical follow-up examinations were performed at baseline (T0) and 12 months postoperatively (T1). During the healing phase, the patient underwent regular maintenance visits for oral hygiene and periodontal assessment.

The primary outcome was horizontal bone thickness (HBT) obtained through alveolar ridge preservation (ARP) using L-PRF and CTG. Secondary outcomes included the changes in PPD, GT and KT as well as patient-reported outcome measures (PROMs) assessing postoperative comfort: number of analgesics taken during the first postoperative week, self-reported pain, Overall Treatment Satisfaction (OTS), and Patient-Related Esthetic Score (PRES).

#### 2.2.1. Radiographic Measurements

##### Image Alignment and Standardization Protocol

For longitudinal assessment, CBCT volumes were standardized through a voxel-based reorientation protocol to ensure reproducibility between T0 and T1. All datasets were reoriented using a three-dimensional coordinate system grounded on the anatomy of the hard palate, selected as a stable intra-subject reference across time.

Specifically, multiplanar reconstructions were adjusted as follows: the median sagittal plane was aligned with the palatal midline bisector, defined by the line connecting the anterior nasal spine (ANS) and posterior nasal spine (PNS); the axial plane was oriented parallel to the palatal plane; and the coronal plane was automatically derived as orthogonal to both sagittal and axial planes. This approach ensured consistent spatial orientation and maximized voxel correspondence across different time points. In the sagittal view, the palatal midline (ANS–PNS axis) provides a reliable anatomical guide for correcting head pitch and defining the plane of symmetry. Finally, in the coronal view, once sagittal and axial orientations are standardized, the coronal plane, set perpendicular to both, benefits from the bilateral symmetry of the palatal vault, allowing correction of roll deviations and ensuring comparability of left–right measurements.

This validation step ensured the spatial superimposability of the analyzed sections and confirmed the reliability of the comparative measurements.

**Figure 1:**
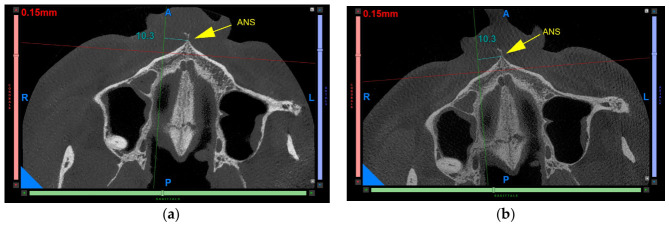
The 10.3 mm distance indicates the patient-specific coordinate from the anterior nasal spine (ANS) to the sagittal section corresponding to the treated site at baseline, which was used to reproduce the same section at the 12-month follow-up. (**a**) Baseline; (**b**) 12-month follow-up.

All analyses were performed using dedicated imaging software NNT-NewTom^®^(version 16.11; Cefla, Imola, Italy) (Figure 1a,b).

##### Morphometric Analysis and Linear Measurements

After reorientation of the CBCT volumes as described above, the sagittal section corresponding to the treated site was identified using the ANS as a stable anatomical landmark. At baseline, the distance between the ANS and the reference axis was 10.3 mm; this value corresponded to the patient-specific sagittal section passing through the center of the extracted tooth/alveolar defect. Therefore, the 10.3 mm distance was used as an individualized anatomical coordinate to standardize sagittal section positioning and ensure reproducibility of the measurements over time. At the 12-month follow-up, the same reference distance was applied to identify the corresponding sagittal section passing through the center of the implant.

**Figure 2:**
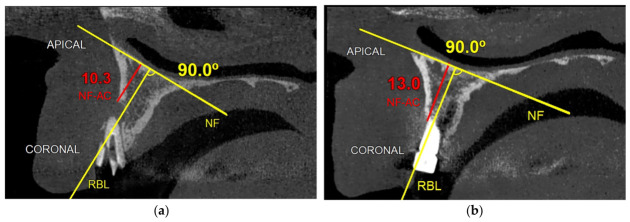
Standardized sagittal CBCT sections for vertical morphometric assessment at baseline (T0; **a**) and 12-month follow-up (T1; **b**). The nasal floor (NF) was used as the anatomical reference; the reference baseline (RBL) was drawn perpendicular to the NF tangent, and the nasal floor–alveolar crest distance (NF–AC) was measured along the RBL.

Moreover, to standardize the measurements, the reference baseline (RBL) was positioned perpendicular to a line tangent to the nasal floor (NF). The vertical measurement, corresponding to the vertical extent of the dehiscence, was defined as the distance between the most coronal point of the buccal bone and the line tangent to the nasal floor, measured along the RBL (Figure 2a,b).

**Figure 3:**
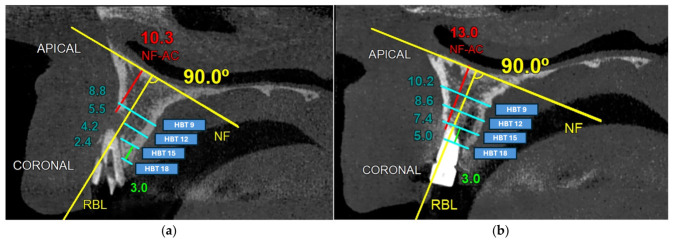
CBCT-based morphometric analysis at baseline (T0) and 12-month follow-up (T1). Horizontal bone thickness (HBT) was measured in the bucco-palatal direction at four apico-coronal levels 9, 12, 15, and 18 mm along the reference baseline (RBL). The nasal floor (NF) was used as a stable anatomical reference, and the nasal floor–alveolar crest distance (NF–AC) was assessed along the RBL. (**a**) Baseline tooth/socket site; (**b**) 12-month implant site.

HBT was measured in the bucco-palatal direction at four predefined apico-coronal levels along RBL, namely 9, 12, 15, and 18 mm on the reference scale, progressing from the apical to the coronal aspect of the ridge (Figure 3a,b). These levels were selected to provide a standardized morphometric description of the apico-coronal pattern of dimensional changes within the treated post-extraction site and to allow reproducible comparison between T0 and T1 using the same reference system. The most coronal level was considered the most clinically relevant from an implant-prognostic perspective, as it better reflects the buccal bone support adjacent to the implant platform and the peri-implant soft-tissue contour. Conversely, the more apical levels were included to characterize the overall remodeling pattern of the residual alveolar/socket region and should not be interpreted as direct evidence of complete buccal bone regeneration around the implant (Figure 3a,b). The measurement strategy was defined in accordance with previously published CBCT-based morphometric approaches for immediate implant evaluation by Jin et al. [17].

#### 2.2.2. Clinical Measurements

- PPD: Measured at baseline as the distance in millimeters from the gingival margin to the bottom of the periodontal pocket and was recorded at six sites around tooth 1.2: mesiobuccal, midbuccal, distobuccal, mesiopalatal, midpalatal, and distopalatal. At the 12-month follow-up, peri-implant probing depth was defined as the distance from the peri-implant mucosal margin to the bottom of the peri-implant sulcus/pocket and was recorded at the corresponding six sites around the implant-supported restoration.
- GT: Measured using the bone sounding technique with a #15 K-file inserted until bone contact. The distance between the file tip and a silicone stop (fixed with cyanoacrylate) was measured using a digital caliper (ABSOLUTE Digimatic Caliper; model 500-196-30; Mitutoyo Corporation, Kawasaki, Japan; accuracy: ±0.02 mm). The measurement point was determined using a custom-fabricated resin stent supported on the adjacent teeth, incorporating a guide hole positioned 3 mm apical to the gingival margin of the tooth of interest prior to extraction. The same stent was consistently employed at T1 to ensure reproducibility of the measurements.
- KT: Measurements were obtained using a UNC-15 periodontal probe, from the most apical portion of the gingival margin to the mucogingival junction, on the tooth at T0 and on the implant at T1.
- PROMs: The patient reported the number of analgesics taken during the first postoperative week and pain levels, assessed using a Visual Analog Scale (VAS). At the 12-month follow-up, esthetic satisfaction was evaluated using the PRES [18] assessed with a VAS based on standardized preoperative (T0) and postoperative (T1) photographs. The OTS was assessed by asking whether the patient would undergo the procedure again, considering the esthetic outcome and perceived pain (yes/no).

### 2.3. Pre-Surgical Phase

Before surgery, the patient received oral hygiene instructions, supragingival scaling, and professional polishing. The use of an electric toothbrush with pressure control and ultra-soft bristles was recommended. At the two-week re-evaluation, T0, clinical measurements were recorded.

### 2.4. Surgical Technique

**Figure 4:**
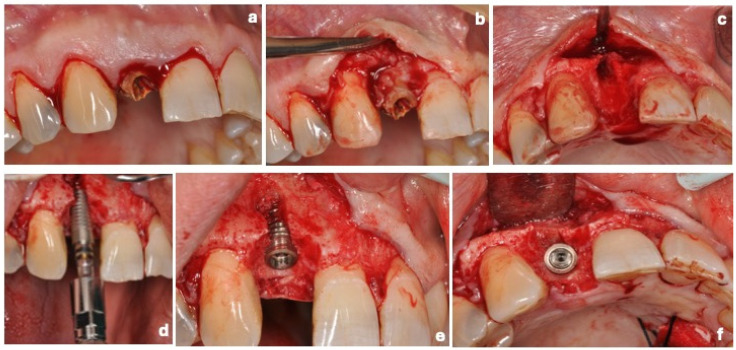
(**a**–**f**) Surgical procedure. (**a**) Preoperative clinical view showing the fractured maxillary lateral incisor 1.2. (**b**) Full-thickness flap elevation clearly exposing the pronounced buccal dehiscence defect. (**c**) Occlusal view after atraumatic extraction and thorough debridement, highlighting the extent of the buccal defect. (**d**) Implant insertion. (**e**) Immediate implant placement achieving primary stability despite the buccal dehiscence. (**f**) Occlusal view of the surgical site, illustrating the extent of the buccal defect following implant placement.

After achieving adequate local anesthesia with 4% articaine and 1:100,000 epinephrine, an envelope intrasulcular flap was performed extending to adjacent teeth, without vertical releasing incisions, to preserve the interdental papillae and optimize vascularization (Figure 4a).

A full-thickness flap was raised to expose the buccal bone defect without surpassing the mucogingival junction (Figure 4b,c). Atraumatic extraction of tooth 1.2 was performed to preserve the residual alveolar walls and minimize trauma. Thorough curettage of the periapical lesion was performed to eliminate the granulation tissue. A transmucosal implant (3.8 × 13 mm way Mix Geass^®^, IESS Group S.r.l., Pozzuolo del Friuli, UD, Italy) was placed following the manufacturer’s protocol, achieving primary stability with an insertion torque >35 N/cm. A delayed loading protocol was adopted (Figure 4d–f).

#### 2.4.1. L-PRF Membrane Preparation

**Figure 5:**
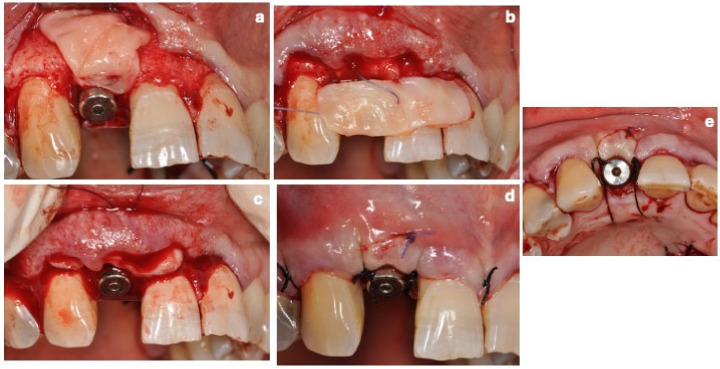
(**a**–**e**) Grafting and soft tissue management. (**a**) Placement of the L-PRF membranes over the buccal aspect of the implant site to compensate for the dehiscence defect. (**b**) Placement and stabilization of the connective tissue graft (CTG). (**c**) Adaptation of the flap over the grafted area. (**d**) Suturing and primary closure of the surgical site. (**e**) Occlusal view illustrating the final stabilization of the soft tissues.

L-PRF membranes were prepared according to Choukroun’s protocol [12]. Thirty milliliters of venous blood were drawn into three 10 mL tubes without anticoagulant and centrifuged at 3000 rpm, corresponding to approximately 503× *g* at clot level, for 10 min (IntraSpin™, Intra-Lock System Europa SpA, Salerno, Italy). The resulting fibrin clots were compressed in an L-PRF Box (Xpression™ Kit, BioHorizons Implant Systems Inc., Birmingham, AL, USA) for 120 s under ~100 g pressure. Two membranes were superimposed to achieve a ~2 mm thickness and inserted into the defect, while a third was positioned over the buccal bone defect (Figure 5a).

#### 2.4.2. Connective Tissue Graft Harvesting

Two horizontal and two vertical incisions were made on the palatal mucosa distal to the upper premolars. A partial-thickness flap was elevated, leaving the periosteum intact. The harvested graft was de-epithelialized extra-orally to achieve ~1 mm thickness and shaped to fit the recipient site, where it was placed to stabilize the flap and augment vestibular soft tissue thickness. The flap was repositioned for complete coverage and secured with 5-0 PGA resorbable sutures (Hu-Friedy, Chicago, IL, USA) (Figure 5b–e).

### 2.5. Postoperative Management

Postoperative management included antibiotic therapy (amoxicillin/clavulanic acid 1 g every 12 h for 6 days) and ketoprofen 80 mg as needed. Chemical plaque control was performed using 0.20% chlorhexidine mouth rinses (Dentosan 0.20, Johnson & Johnson, New Brunswick, NJ, USA) and topical gel with chlorhexidine 1% (Corsodyl gel, GlaxoSmithKline Consumer Healthcare, Brentford, UK), both prescribed twice daily for 15 days. The patient was instructed to avoid brushing or trauma at the surgical site for at least 4 weeks. Follow-up appointments were planned at 1 and 2 weeks for suture removal, monthly during the first 6 months, and at 12 months (T1) for the final clinical and radiographic assessment.

## 3. Results

### 3.1. Radiographic Analysis

Radiographic evaluation revealed measurable dimensional changes between T0 and T1, indicating a favorable hard tissue response at the treated site.

HBT demonstrated a consistent increase across all evaluated apico-coronal levels. Specifically, HBT increased from 2.4 mm to 5.0 mm at 18 mm (+2.6 mm), from 4.2 mm to 7.4 mm at 15 mm (+3.2 mm), from 5.5 mm to 8.6 mm at 12 mm (+3.1 mm), and from 8.8 mm to 10.2 mm at 9 mm (+1.4 mm) (Figure 3a,b).

The NF–AC increased from 10.3 mm at T0 to 13.0 mm at T1, corresponding to a gain of +2.7 mm. Furthermore, the buccal dehiscence present at baseline appeared to be markedly reduced at the 12-month evaluation, suggesting substantial defect resolution (Figure 2a,b).

Overall, the radiographic findings indicate a non-uniform pattern of bone remodeling, characterized by greater dimensional gains in the mid-apical region of the ridge, whereas less change was detected at the coronal level. This pattern is consistent with a favorable regenerative response, with improved ridge dimensions and partial restoration of the buccal bone profile (Table 1).

### 3.2. Clinical Outcomes and PROMs

Clinical healing was uneventful at both the recipient and palatal donor sites. No postoperative infection, wound dehiscence, suppuration, bleeding, or functional impairment was observed.

**Figure 6:**
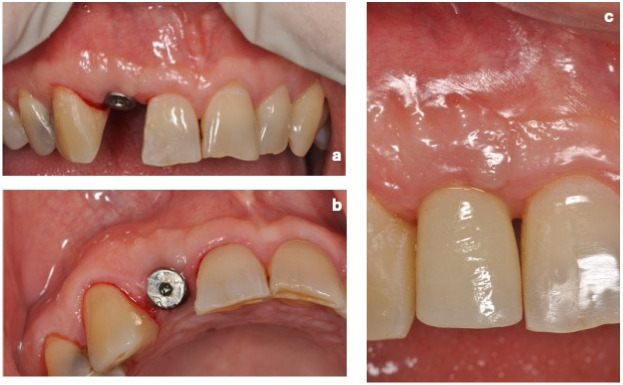
(**a**–**c**) Clinical outcomes at 12-month follow-up. (**a**) Frontal view showing stable peri-implant soft tissues and favorable esthetic integration. (**b**) Occlusal view demonstrating adequate contour and thickness of the peri-implant tissues. (**c**) Final clinical view after placement of the definitive crown, highlighting harmonious integration with the surrounding dentition and stable peri-implant soft tissue conditions.

Throughout the 12-month follow-up period, the peri-implant tissues remained clinically healthy, with no signs of inflammation, mucositis, or peri-implant bone loss. The implant was loaded 6 months after surgery (Figure 6a–c).

**Figure 7:**
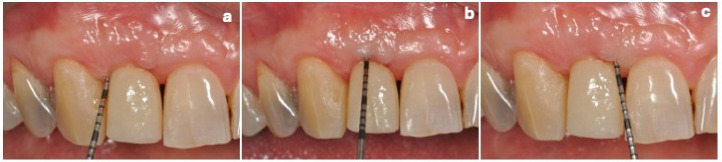
(**a**–**c**) Clinical probing assessment at 12-month follow-up. (**a**–**c**) Periodontal probing of the buccal aspect of the implant site demonstrating the absence of probe penetration, despite incomplete radiographic buccal bone coverage. This finding suggests the presence of a stable soft tissue seal.

At the 12-month follow-up, the implant was clinically stable and functional. Full peri-implant probing assessment showed probing depths of 3 mm at the mesiobuccal site, 4 mm at the midbuccal site, 3 mm at the distobuccal site, 4 mm at the mesiopalatal site, 3 mm at the midpalatal site, and 4 mm at the distopalatal site. No bleeding on probing was observed (Figure 7a–c).

**Figure 8:**
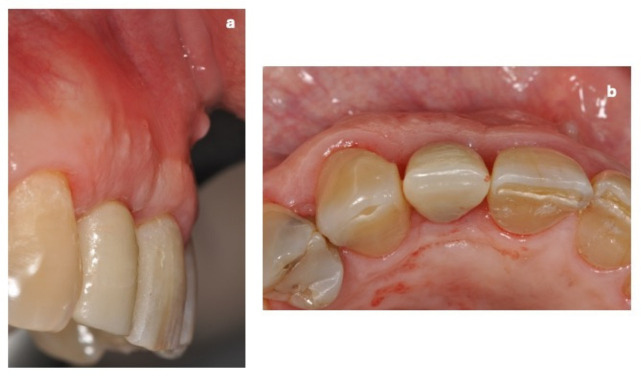
(**a**,**b**) Soft tissue augmentation outcome at follow-up. (**a**) Lateral view highlighting the increased thickness of the peri-implant soft tissues following CTG placement. (**b**) Occlusal view demonstrating improved soft tissue volume and contour, reflecting the effectiveness of the soft tissue augmentation procedure.

Regarding soft tissue parameters, the KT increased from 3.0 mm to 5.0 mm, with a gain of +2.0 mm, while the GT increased from 1.4 mm to 3.2 mm, with a gain of +1.8 mm (Figure 8a,b; Table 1).

The patient assumed only two doses of ketoprofen 80 mg during the two days following the surgical procedure. Thereafter, no further analgesics were required.

Pain was reported only on the first postoperative day (VAS = 7), decreased substantially on the second day (VAS = 3), was almost absent by the third day (VAS = 1), and completely resolved by the fourth day.

With regard to PRES, the subject assigned the maximum score (VAS = 10), stating that—based on the minimal discomfort experienced—he would undergo the same surgical procedure again (OTS: yes).

## 4. Discussion

This clinical report documented favorable 12-month clinical and radiographic outcomes following a fully autologous ARP protocol combining L-PRF and CTG with immediate implant placement for the management of a post-extraction socket with buccal dehiscence in the esthetic zone. The proposed approach was designed to enhance biologic healing while avoiding the use of heterologous biomaterials and reducing surgical complexity.

A standardized CBCT-based protocol enabled reproducible assessment of both horizontal and vertical dimensional changes. The use of stable anatomical landmarks, fixed reference planes, and multi-level measurements allowed consistent site matching over time and was in line with previously validated methodologies [17], supporting the reliability of the present findings.

The radiographic analysis revealed measurable dimensional changes between baseline and the 12-month follow-up, indicating a favorable hard tissue response. HBT increased at all evaluated apico-coronal levels, with gains of +2.6 mm at 18 mm, +3.2 mm at 15 mm, +3.1 mm at 12 mm, and +1.4 mm at 9 mm. Greater dimensional increases were observed in the mid-apical part of the ridge.

NF-AC measurements showed an increase of +2.7 mm, together with a marked reduction in the buccal dehiscence defect.

This radiographic pattern is consistent with the physiological dynamics of post-extraction remodeling, which predominantly affect the coronal portion of the alveolar ridge as a consequence of bundle bone resorption and facial plate remodeling [19].

In the present case, the relative preservation of the coronal ridge contour, together with the marked radiographic reduction in the buccal dehiscence defect, may suggest modulation of the expected post-extraction resorptive pattern and localized radiographic hard-tissue reconstitution [2,20].

The mid-apical radiographic changes observed at 12 months should be interpreted within the broader multifactorial healing process following tooth extraction. The apical area of the socket is generally less affected by the pronounced dimensional changes that occur coronally after extraction and may retain a more favorable biological environment, characterized by improved clot stabilization, greater wound stability, availability of bone-derived cells, and vascular support from residual socket walls and basal bone [20]. However, these findings should be interpreted as CBCT-based radiographic changes and cannot be considered definitive histological evidence of new bone formation.

The biologic rationale underlying the present protocol is based on the complementary roles of L-PRF and CTG. The L-PRF provides a fibrin matrix that supports cell migration and angiogenesis through the sustained release of growth factors, thereby promoting early wound stability [11,21]. In parallel, CTG contributes to mechanical stabilization of the healing site and enhances soft tissue thickness, which is critical in preventing collapse of the facial profile. This combined effect is particularly relevant in compromised sites, where both vascular supply and tissue support are key determinants of regenerative outcomes [6,22].

The observed increase in KT and GT further supports the clinical relevance of the proposed approach from a peri-implant soft-tissue perspective. Adequate peri-implant soft-tissue dimensions have been associated with improved plaque control, reduced mucosal inflammation, and greater marginal bone stability [6,7]. In this context, the use of CTG in conjunction with immediate implant placement may contribute to improving the peri-implant soft-tissue phenotype, including tissue thickness and keratinized tissue width, both of which are considered important determinants of long-term biological and esthetic stability [6,7,23,24,25] (Figure 8a,b). In particular, a keratinized tissue width of at least 2 mm has been suggested to be beneficial for peri-implant tissue health, especially in esthetically demanding areas [26,27,28]. Similarly, increased soft-tissue thickness has been associated with a protective effect against crestal bone resorption over time [26,27,28,29,30].

Nevertheless, although CTG may contribute to increasing peri-implant soft-tissue thickness, preserving mucosal architecture, and reducing the risk of buccal soft-tissue dehiscence, the absence of a buccal bone wall remains an important anatomical limitation.

From a clinical perspective, this condition may raise concerns regarding long-term esthetic and biological stability, including possible soft tissue discoloration, future mucosal recession, unfavorable biomechanical conditions, and peri-implant inflammatory complications. In the present case, no such complications were observed at the 12-month follow-up; however, longer-term clinical and radiographic monitoring is required to confirm the stability of the observed outcome [31].

Notably, in this case report, the CBCT evaluation suggested that, although the NF–AC distance increased after treatment, the crestal bone level did not reach the most coronal portion of the implant, and it was not completely covered by bone, as shown in Figure 3b. This incomplete coronal buccal bone coverage represents an important anatomical limitation and requires careful long-term clinical and radiographic monitoring. It may therefore be hypothesized that the placement of autogenous or xenogeneic bone graft material beneath the L-PRF membranes and CTG could have provided additional space-maintaining support, potentially contributing not only to the modulation of buccal bone resorption, but also to greater HBT values, including at the level of the implant platform.

Nonetheless, the implant was not clinically probeable (Figure 7a–c). This finding may suggest that the increased soft-tissue thickness and quality of the connective tissue graft contributed to a favorable mucosal seal, potentially limiting bacterial infiltration despite incomplete radiographic bone coverage [23,26].

Although immediate implant placement may contribute to partial preservation of socket architecture, it does not prevent physiologic resorption of the facial bone plate, particularly in the presence of pre-existing defects [20]. Therefore, in such clinical cases, adjunctive strategies aimed at stabilizing the wound environment and enhancing peri-implant soft-tissue architecture may be important for maintaining the ridge contour over time [32].

From a mechanistic standpoint, the present approach shows conceptual similarities with the PI strategy [15], which aims to limit external bone resorption by modulating osteoclastic activity originating from the periosteal compartment. In this context, CTG may have acted as the main autologous interpositional component of the protocol, potentially contributing to soft-tissue thickening, mucosal sealing, phenotype modification, and stabilization of the treated site.

When positioned in close contact with the facial bone, CTG may theoretically contribute to wound stabilization and soft-tissue sealing, thereby supporting facial contour maintenance after immediate implant placement. Accordingly, the present protocol may be interpreted as a biologically oriented autologous ARP strategy for carefully selected clinical scenarios.

Conversely, L-PRF membranes were not intended to function as long-lasting occlusive membranes or primary space-maintaining devices. Rather, their role was mainly biological, providing a fibrin-based matrix intended to support clot stabilization, angiogenesis, and early wound healing through the sustained release of growth factors.

It should be emphasized that the present protocol differs conceptually from conventional GBR and should not be interpreted as an attempt to reproduce its mechanical and barrier-related functions. Rather, the proposed approach may be considered in selected sites presenting with mild-to-moderate buccal dehiscence defects, where soft-tissue support, wound stability, and vascularization are critical determinants of healing.

Conventional GBR remains a well-established approach for the reconstruction of peri-implant and alveolar ridge defects, particularly when the clinical objective is to create and maintain a protected regenerative compartment. Its biological rationale is based on primary wound closure, angiogenesis, space creation and maintenance, and stabilization of the initial blood clot [33,34]. Barrier membranes also provide selective cell exclusion by limiting the ingress of rapidly migrating epithelial and connective tissue cells while allowing osteogenic cells to repopulate the defect area [33,35]. These properties are particularly relevant in non-contained defects, where mechanical stability and prolonged protection of the regenerative space are essential for treatment predictability.

In contrast, the present protocol may be regarded as a biologically oriented, fully autologous ARP strategy aimed at supporting early wound stability, peri-implant soft-tissue phenotype modification, and preservation of the facial ridge contour in carefully selected clinical cases [9,36]. These features may be relevant in selected esthetic scenarios, particularly when soft-tissue volume, mucosal stability, and patient-specific considerations favor an autologous, biomaterial-sparing strategy.

This interpretation is partially consistent with previous clinical studies suggesting that CTG does not necessarily promote new bone formation but may contribute to preserving existing alveolar bone [26,29]. However, the combination of L-PRF and CTG provides limited mechanical space maintenance, lacks a prolonged rigid barrier function, and may be less predictable in extensive or poorly contained buccal defects. Moreover, CTG harvesting increases surgical morbidity and technique sensitivity, and the individual contribution of L-PRF, CTG, implant positioning, defect morphology, and local healing conditions cannot be isolated in a single case report.

Accordingly, the present approach should be regarded as a biologically oriented autologous strategy, conceptually based on the rationale of PI, for carefully selected clinical situations rather than as a replacement for conventional GBR. 

The present case report is limited by its single-case design and lack of a control group, which preclude definitive conclusions. In addition, although CBCT-based linear measurements are clinically informative, they do not allow discrimination between histologically confirmed bone regeneration and compensatory remodeling. Furthermore, the relatively short 12-month follow-up period does not allow conclusions regarding the long-term stability of peri-implant tissues. Future controlled clinical studies with larger cohorts and extended follow-up periods are required to validate these preliminary findings and define the predictability, indications, and limitations of the proposed technique. 

## 5. Conclusions

Within the limitations of this case report, L-PRF membranes combined with CTG and immediate implant placement were associated with favorable clinical and radiographic outcomes in the management of a post-extraction socket with buccal dehiscence in the esthetic zone. These preliminary observations may be considered consistent with the biological rationale of periosteal inhibition in this clinical scenario.

Increased peri-implant soft-tissue thickness, improved keratinized tissue dimensions, and radiographic maintenance of the treated ridge contour were also observed. 

However, no definitive conclusions can be drawn regarding the predictability of this approach, its comparative effectiveness relative to conventional GBR, or the respective contribution of L-PRF, CTG, implant positioning, defect morphology, and local healing conditions. Further studies involving larger cohorts, standardized radiographic protocols, and extended follow-up periods are required to validate these preliminary observations.

## Figures and Tables

**Table 1:** Radiographic and clinical outcomes measured at baseline (T0) and 12 months (T1), with corresponding dimensional gains.

Parameters	Baseline	12 Month Follow Up	Gain
**Radiographic outcomes**			
HBT 18 mm	2.4 mm	5.0 mm	2.6 mm
HBT 15 mm	4.2 mm	7.4 mm	3.2 mm
HBT 12 mm	5.5 mm	8.6 mm	3.1 mm
HBT 9 mm	8.8 mm	10.2 mm	1.4 mm
NF-AC	10.3 mm	13.0 mm	2.7 mm
**Clinical Outcomes**			
KT	3 mm	5 mm	2 mm
GT	1.4 mm	3.2 mm	1.8 mm
**PPD**	**Tooth**	**Implant**	**Variation**
Mesiobuccal	4 mm	3 mm	−1 mm
Midbuccal	15 mm	4 mm	−11 mm
Distobuccal	5 mm	3 mm	−2 mm
Mesiopalatal	3 mm	4 mm	+1mm
Midpalatal	3 mm	3 mm	0 mm
Distopalatal	3 mm	4 mm	+1 mm

## Data Availability

The original contributions presented in this study are included in the article. Further inquiries can be directed to the corresponding author.
